# Radial longitudinal deficiency: long-term outcomes of radialization and vascularized metatarsophalangeal joint transfer

**DOI:** 10.1177/17531934251391119

**Published:** 2025-11-11

**Authors:** Niko Kämppä, Petra Grahn, Pasi Paavilainen, Yrjänä Nietosvaara, Jarkko Jokihaara

**Affiliations:** 1 Department of Hand Surgery, HUS Helsinki University Hospital, Hyvinkää Hospital, and University of Helsinki, Helsinki, Finland; 2 Department of Paediatric Orthopaedics and Traumatology, University of Helsinki and Helsinki University Hospital, Helsinki, Finland; 3 Pihlajalinna Hospital, Tampere, Finland; 4 Department of Pediatric Surgery, Kuopio University Hospital, University of Eastern Finland, Kuopio, Finland; 5 Division of Musculoskeletal Diseases, Tampere University Hospital, Tampere, Finland; 6 Faculty of Medicine and Health Technology, Tampere University, Tampere, Finland

**Keywords:** Microvascular metatarsophalangeal joint transfer, radial longitudinal deficiency, radial club hand, radialization, Vilkki procedure

## Abstract

**Introduction:**

We studied long-term outcomes of radialization and free vascularized second metatarsophalangeal joint transfer for radial longitudinal deficiency using patient-reported and objective outcome measures.

**Methods:**

Patients aged ⩾10 years with Bayne and Klug type III or IV radial longitudinal deficiencies were identified from national referral centres. Patients treated by radialization (*n* = 15 limbs) and metatarsophalangeal joint transfer (*n* = 17 limbs) were assessed after a median follow-up of 13 years.

**Results:**

The respective scores after metatarsophalangeal joint transfer and radialization were: median Disabilities of Arm, Shoulder, and Hand scores 19 (95% CI: 7 to 25) and 19 (16 to 29); patient-rated wrist evaluation scores 11 (95% CI: 8 to 22) and 24 (95% CI: 10 to 38); and satisfaction with cosmesis 5 (95% CI: 3 to 6) and 8 (95% CI: 7 to 10). Median wrist active range of motion values were 90° (95% CI: 70 to 90) and 50° (95% CI: 30 to 60) and median wrist deviations were 20° (95% CI: 15 to 30) and 30° (95% CI: 15 to 60) after joint transfer and radialization, respectively. Secondary wrist procedures were more frequent in the joint transfer group.

**Conclusions:**

Both techniques yield good functional outcomes. Joint transfer produced a more consistent and larger range of active wrist extension–flexion but with poorer cosmetic results than radialization.

**Level of evidence:**

IV

## Introduction

Radial longitudinal deficiency (RLD) is the most common congenital longitudinal deficiency with an estimated incidence of 1:6100–20 000 live births (Ekblom et al., 2010; Koskimies et al., 2011). It is a spectrum of malformations affecting the structures of the radial side of the forearm, including hypoplasia of the bones, joints and soft tissues (James et al., 1999; Van Nieuwenhoven et al., 2023). The Bayne and Klug classification system (Bayne and Klug, 1987) categorizes forearm deformities into types I–IV based on skeletal differences. However, this system does not account for the often severe soft tissue involvement, which contributes significantly to the condition (James et al., 1999; Van Nieuwenhoven et al., 2023).

Soft tissue stretching and splinting starting immediately after birth seem to benefit all patients, but further treatment of progressive deformity is difficult and the optimal treatment strategy remains unclear (Colen et al., 2017; Murphy et al., 2017; Van Nieuwenhoven et al., 2023). Traditionally the goal of treatment has been a straight wrist (Colen et al., 2017; Ezaki, 2021; Murphy et al., 2017; Van Nieuwenhoven et al., 2023). To achieve this, centralization procedures have been widely used. To improve results, Buck-Gramcko (1985) modified the technique and termed this procedure radialization. However the shortcomings of both centralization and radialization include recurrence of deformity, stiffness and impaired ulnar growth (Murphy et al., 2017). To address these, a free vascularized second toe metatarsophalangeal (MTP) joint transfer was developed (Vilkki, 2008). Improved ulnar length and active range of motion (AROM) of the wrist with minimal donor site morbidity during growth have been reported with this technique (Hellevuo et al., 2023; Murphy et al., 2017; Vilkki and Paavilainen, 2018), but recurrence of radial deviation can be expected (Vilkki and Paavilainen, 2018).

The traditional dogma of striving towards a straight wrist has been challenged, as wrist malposition does not seem to be the most important factor causing disability (Ekblom et al., 2013, 2014; Ezaki, 2021; Goldfarb et al., 2002). Despite wrist deviation and impaired AROM, children adapt and perform daily activities well, making the emphasis on wrist alignment and traditional objective measures (e.g. wrist angulation or AROM) questionable (Buffart et al., 2008). There is limited knowledge about patients’ own assessment of their hand function in RLD and the outcomes of different treatments (Buffart et al., 2008; Ekblom et al., 2014; Goldfarb et al., 2002; Kotwal et al., 2012). The aim of this study was to report long-term objective and patient-reported outcome measures (PROMs) after radialization and free vascularized MTP-transfer in RLD.

## Methods

### Patients

All patients aged 10 years or more with Bayne and Klug type III or IV RLD who underwent operative treatment at Tampere or Helsinki University Hospitals and had accessible medical records were identified. These tertiary units provide centralized RLD care for the entire national population. Only patients who had undergone radialization or MTP-transfer procedures were included in the study. Treatment type was determined by geographical catchment areas: patients in Tampere underwent an MTP-transfer and patients in Helsinki a radialization procedure.

Medical records were reviewed for RLD type, associated conditions, all treatments and complications. Postoperative infections were classified as complications if they required intravenous antibiotics, hospitalization or surgery. Outcomes were assessed with clinical examination by specialists in hand surgery, radiographs and PROM questionnaires. Radialization patients were assessed by NK, uninvolved in previous treatment, and MTP-transfer patients were assessed by PP, who had participated in their earlier care.

Nineteen operatively treated RLD patients were identified at the Helsinki University Hospital. Of these, one patient was treated with a fibular graft reconstruction and was excluded and one was deceased. The remaining 17 had undergone radialization and were invited to participate. One was excluded owing to severe cognitive disability, one declined, and two were unreachable, leaving 13 participants with 15 operated limbs for the study. Eighteen operatively treated RLD patients were identified at the Tampere University Hospital. Two patients declined to participate resulting in 16 participants with 17 operated limbs (Table 1). One patient with bilateral RLD underwent a radialization in Helsinki, and after moving to a different area, the contralateral side was operated with MTP-transfer in Tampere, and the outcome measurements of each of the limbs were included in the corresponding treatment groups.

**Table 1.** Demographic and clinical characteristics of participants. Bilaterally afflicted and operated patients (*n* = 4) are displayed limb-wise as two individual participants in this table.

	Radialization	MTP transfer
	(*n* = 15)	(*n* = 17)
Bayne and Klug classification		
Type III	3	7
Type IV	12	10
Sex		
Male/female	13/2	10/7
Treated side		
Right/left	10/5	8/8
Data on affected side missing	0	1
Bayne and Klug classification of contralateral side		
Normal	9	14
Type I	1	0
Type III	1	0
Type IV	4	3
Age at operation (months)		
Median (IQR)	17.1 (7.8–24.0)	28.0 (16.6–32.0)
Age at follow-up (years)		
Median (IQR)	13.6 (11.4–19.6)	16.8 (14.2–23.7)
Follow-up time (years)		
Median (IQR)	12.4 (9.8–16.5)	13.5 (11.8–21.2)

MTP: Metatarsophalangeal joint.

### Surgical interventions

In both procedures, initial soft tissue distraction was done to restore hand alignment. The technique for microsurgical MTP-transfer has been described previously in detail (Vilkki, 2008). Four type III patients subsequently underwent an additional reconstruction of a two-bone forearm with a neo-radius, in which the MTP-graft was detached from the ulna and reattached to the proximal radius (Vilkki and Paavilainen, 2018). Radialization procedures were done following the description of Buck-Gramcko (1985), with some variations to accommodate individual anatomical differences. Three patients underwent procedures resembling centralization, which excluded tendon transfers and involved partial resection of carpal bones or the ulna (Manske et al., 1981). Surgical interventions were carried out between the years 1984 and 2017.

### Objective and patient reported outcome measures

Wrist deviation at rest, flexion at rest and AROM of extension–flexion were measured with a dorsally placed goniometer. Forearm rotation was measured with the elbow flexed to 90°, the arm adducted, and the wrist stabilized into a position as straight as possible.

Patients completed the Patient-rated Wrist Evaluation (PRWE) and the Disabilities of Arm, Shoulder, and Hand (DASH) questionnaires. The cross-culturally validated Finnish versions of both PROMs were used (Aro et al., 2009; Sandelin et al., 2016). Since the DASH does not differentiate between sides, participants with bilateral RLD received the same score for both extremities. Overall satisfaction with function and cosmesis was assessed with a numerical rating scale (NRS) from 0 to 10 with higher scores indicating greater satisfaction.

### Radiographic measurements

Standard posteroanterior and lateral radiographs of both forearms and hands were obtained with the affected wrist positioned as straight as possible. Complete sets of radiographs were not available for three participants after the MTP-transfer. The hand–forearm angle (HFA), hand–forearm position (HFP), ulnar length (UL) and ulnar bow (UB) were measured as proposed by Manske et al. (1981). Total carpal forearm length (TCFL), total forearm angle (TFA) and the modified hand–forearm position (mHFP) were measured as described by Ekblom et al. (2014). In participants with unilateral presentation UL was compared with the length of the unaffected ulna. The contralateral side was considered to be unaffected if there was no evident shortening of the radius either clinically or radiographically (James et al., 1999).

### Statistical analysis

We present results of MTP-transfers and radialization with median and 95% confidence intervals (CIs) or where specifically indicated with median and interquartile range (IQR). Confidence intervals were obtained using a nonparametric basic bootstrapping method owing to the heavy skew in distributions. Spearman’s rank correlation was used to explore possible correlation of age, wrist position and AROM with outcome measures. A correlation with a coefficient of <0.4 was considered weak, 0.4–0.59 moderate and >0.6 strong. Formal comparative statistical analyses between treatment groups were not done because these would be likely to be untrustworthy and potentially misleading in a small study population.

## Results

**Figure 1.**
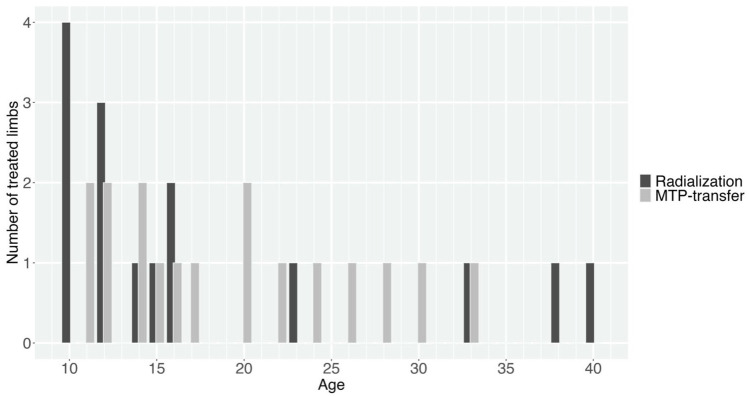
Age (at follow-up) distribution of participants in the radialization and microvascular metatarsophalangeal joint (MTP) transfer groups. Participants with bilateral radial longitudinal deficiency (RLD) and both sides operatively treated (*n* = 4) are included by limb in this histogram.

Nineteen of the 28 participants were male. Radial longitudinal deficiency involvement was bilateral in five participants and both sides were operated on in four participants. VACTERL (vertebral defects, anorectal malformations, cardiac defects, tracheo-esophageal fistula, renal anomalies and limb abnormalities) was the most common associated systemic disorder (six patients in the radialization group; four patients in MTP-transfer group), followed by valproate syndrome (two patients in radialization group), Goldenhar syndrome (one patient in MTP-group) and Klippel–Feil syndrome (one patient with bilateral RLD). Median age at follow-up, follow-up times and clinical characteristics are presented in Table 1. The age distribution of participants is displayed in Figure 1.

The course of treatment for all participants is outlined in Table S1 (available online). Participants in both groups underwent additional procedures unrelated to the MTP-transfer or radialization, with pollicization being the most common (9/15 in the radialization group; 11/17 in MTP-transfer group; Table S1).

### Radialization group

**Figure 2.**
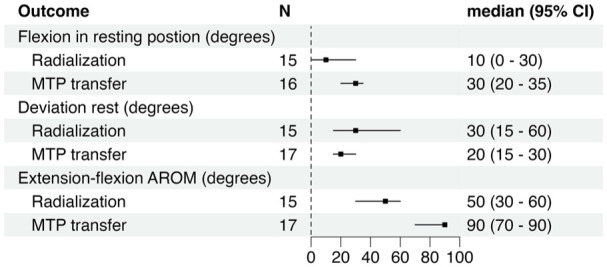
Clinically evaluated wrist position and active range of motion (AROM). Metatarsophalangeal joint (MTP).

**Figure 3.**
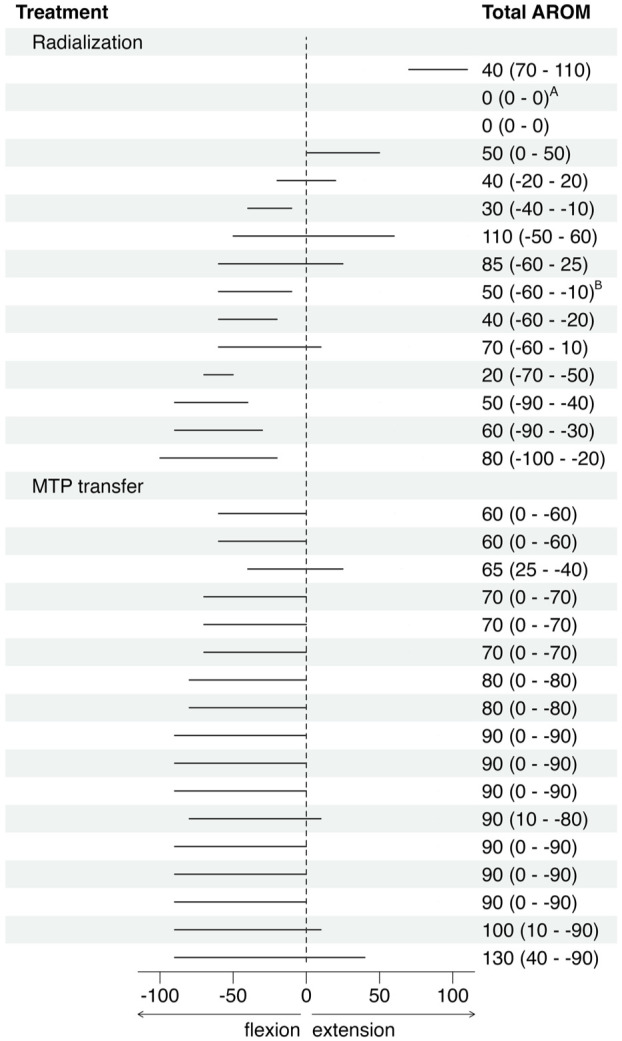
Active wrist extension–flexion range of motion (AROM) per affected limb. Metatarsophalangeal joint transfer (MTP transfer). ^A^Wrist fusion, ^B^failed wrist fusion.

Median wrist extension–flexion and deviation position are displayed in Figure 2. After radialization the wrist position ranged from 65° of flexion to 80° of extension with 6/15 wrists in neutral position at rest, with others displaying different degrees of flexion (range 10–65°) and one in marked extension (80°). Wrist deviation ranged from 0 to 80°. The total extension–flexion AROM ranged from 0 to 110° and the sector where motion was observed varied greatly (Figure 3). Median AROM values are displayed in Figure 2. Median pro-supination AROM was 35° (range 0–85°), with 10/15 extremities showing motion.

Primary complications included an intraoperative distal ulna fracture causing a partial growth arrest, one case of loss of wrist position owing to inadequate K-wire fixation and one K-wire protrusion requiring early removal. Secondary operations were done in three patients, including two ulnocarpal fusions (Table S1, available online).

### MTP-transfer group

Median values of wrist position and AROM are displayed in Figure 2. Wrist position remained consistent after MTP-transfer, with all wrists in slight flexion (range 10–65°) and radial deviation (range 10–45°). Wrist extension–flexion AROM ranged from 60 to 130°. Only two wrists had extension above neutral (Figure 3). A median AROM of 65° (range 40–100°) of forearm rotation was seen in the four limbs that underwent a reconstruction of a neo-radius.

Primary complications included one delayed bony union between the graft and ulna, causing widening of the graft–ulna junction. Secondary operations related to the MTP-transfer were carried out in 6/17 limbs. These included four neo-radius reconstructions (Vilkki and Paavilainen, 2018) (Table S1, available online).

## Objective and patient reported outcome measures

**Figure 4.**
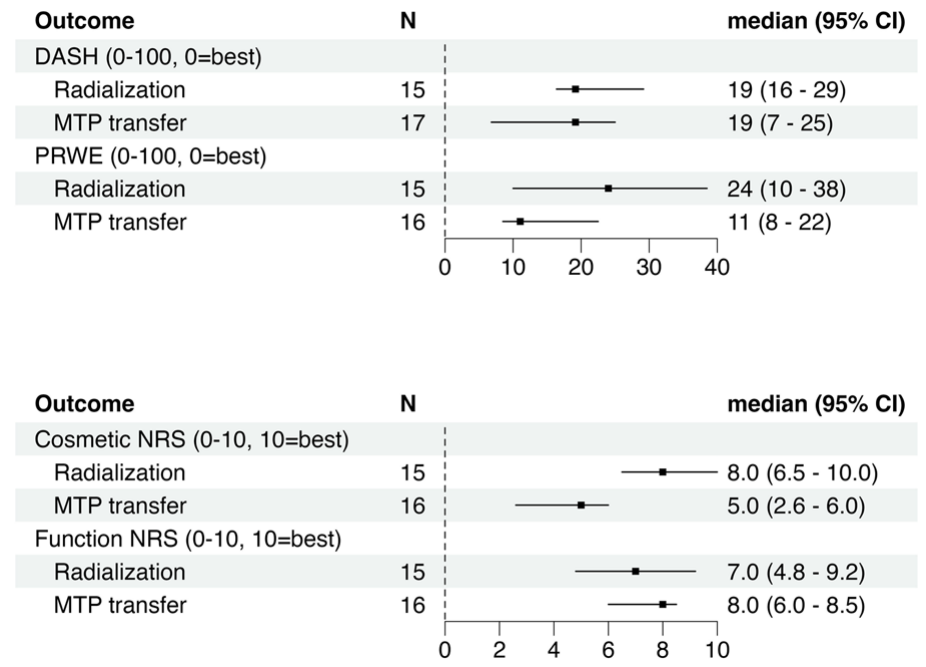
Summary of patient reported outcomes. The Disabilities of the Arm, Shoulder and Hand (DASH), Patient-rated Wrist Evaluation (PRWE), metatarsophalangeal joint (MTP), numeric rating scale (NRS) and active range of motion (AROM).

DASH, PRWE and function NRS scores indicated low disability in both groups (Figure 4). Cosmetic satisfaction seemed lower in the MTP-transfer group (Figure 4).

### Radiographic measurements

**Figure 5.**
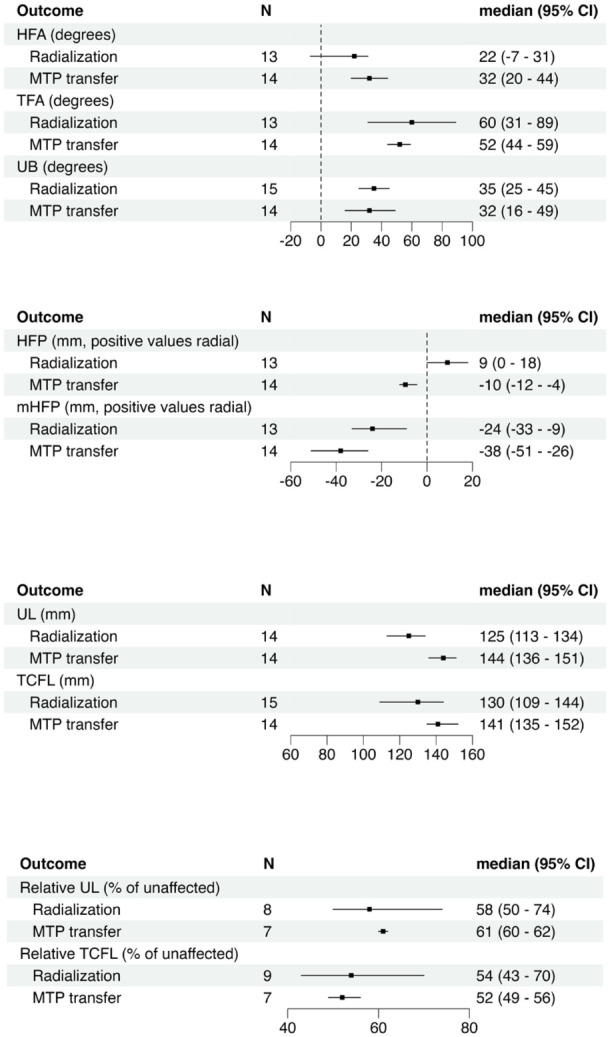
Summary of radiographic outcomes. Metatarsophalangeal joint II (MTP II); numeric rating scale (NRS); hand–forearm angle (HFA), angle between axis of third metacarpal and distal ulna; total forearm angle (TFA), angle between axis of third metacarpal and proximal ulna; ulnar bow (UB), angle between axis of distal and proximal ulna; hand–forearm position (HFP) shortest distance between the longitudinal axis of the distal ulna and the base of the ulnarmost metacarpal; modified hand–forearm position (mHFP), shortest distance between the most proximal point of the base of the small finger metacarpal and the longitudinal axis of the proximal ulna; ulnar length (UL), measured between the midpoint of the proximal and distal ulna; total carpal forearm length (TCFL), shortest distance between the most proximal point of the base of the third metacarpal and the tip of the olecranon. Relative UL and TCFL calculated as percentage of unaffected contralateral side.

Radiological findings for both groups are presented in Figure 5. The direction of deformity after radialization prevented measurement of HFA, HFP, TFA and mHFP from standard radiographs for two participants. Ulnar length could not be measured for one additional participant owing to an ulnocarpal fusion.

### Correlation between age, wrist position and AROM with other outcomes

Spearman’s rank correlations between age and outcomes, HFA and PROMs as well as extension–flexion AROM and PROMs are displayed in Table 2. Correlations of forearm AROM with outcomes were not calculated for MTP-transfers as only four participants had some motion. In the radialization group correlation between AROM of forearm rotation and DASH (*r* = −0.07; *p* = 0.81), PRWE (*r* = −0.24; *p* = 0.37) and function NRS (*r* = −0.16; *p* = 0.57) was statistically insignificant and weak.

**Table 2.** Correlations between age, hand–forearm angle (HFA), active range of motion and other outcomes.

		Radialization		MTP-transfer	
		*r*	*p*-Value	*r*	*p*-Value
Age	DASH	0.28	0.31	0.07	0.80
	PRWE	−0.04	0.89	−0.26	0.32
	Cosmetic NRS	−0.47	0.07	0	0.99
	Function NRS	−0.43	0.10	−0.03	0.92
	Wrist deviation	−0.28	0.31	0.25	0.34
	Wrist flexion deformity	0.24	0.39	0.46	0.07
HFA	DASH	−0.05	0.86	−0.31	0.28
	PRWE	−0.13	0.67	−0.44	0.12
	Cosmetic NRS	0.17	0.58	−0.09	0.77
	Function NRS	0.49	0.09	0.32	0.27
AROM	DASH	−0.02	0.93	−0.43	0.08
	PRWE	−0.52	**0.05**	−0.40	0.13
	Function NRS	0.28	0.32	0.28	0.30

DASH: The Disabilities of the Arm, Shoulder and Hand score; PWRE: Patient-rated Wrist Evaluation; MTP: metatarsophalangeal transfer; NRS: numeric rating scale (NRS); AROM: active range of motion for extension-flexion of wrist. Statistically significant values are shown in bold.

## Discussion

The PROMs indicated similar and relatively mild disability after both radialization and MTP-transfer in RLD. In addition, ulnar length and wrist alignment appear comparable in both treatment groups. However, while MTP-transfer seems to achieve a more consistent and larger AROM of extension-flexion when compared with radialization, this appears to come at the cost of a less favourable aesthetic outcome for the extremity and increased surgical complexity associated with microsurgical reconstruction.

Our wrist extension–flexion AROM results correspond to the pooled data of Murphy et al. (2017) showing a notable reduction particularly after radialization. However, our findings suggest a difference in the motion pattern: MTP-transfers consistently show a wider flexion-dominant arc of motion, whereas radialization outcomes are more variable. In Bayne and Klug type III deformities, MTP-transfer with neo-radius reconstruction can enable forearm rotation movement (Vilkki and Paavilainen, 2018). However a substantial portion of patients in the radialization group also had forearm rotation, although the explanation for this is not clear.

Although statistical significance was limited by the small sample size, the AROM of extension–flexion seemed to correlate moderately with PRWE in both groups and with DASH in the MTP-transfer group. Despite this, the increased AROM after MTP-transfers did not translate into better patient-reported outcomes. Function NRS and PRWE, not previously used in RLD, indicated low and similar disability after both procedures. DASH scores were also similar between groups and consistent with the centralization outcomes reported by Goldfarb et al. (2002). The lack of PROM improvement despite greater wrist mobility after MTP-transfer may reflect the relative importance of finger mobility and grip strength in daily activities (Ekblom et al., 2014; Van Nieuwenhoven et al., 2023). Daily activity needs and related PROM performance can vary by age and wrist deformities tend to recur with growth (Murphy et al., 2017). However, no statistically significant correlations were found between age and PROMs or age and wrist alignment. This is consistent with previous reports in centralization patients (Ekblom et al., 2013, 2014; Goldfarb et al., 2002). The heterogeneity of the severity of RLD and the small sample sizes increase uncertainty in these correlation analyses.

Recurrence of deformity was common regardless of the surgical approach. Wrist deviation measured by HFA was consistent with previously reported pooled data (Murphy et al., 2017). Hand–forearm angle, clinical wrist deviation and flexion malposition were similar after both procedures. Although both treatment groups included adolescents with some growth still to come, there were more adolescents in the radialization group. Therefore, the recurrence of wrist malposition after radialization might be underestimated when compared to MTP-transfers. Wrist alignment measured with HFA did not show statistically significant correlation with patient-reported function. Radialization seemed to result in better cosmetic outcomes. As wrist alignment did not seem to correlate with cosmetic NRS, it seems likely that the more extensive incisions and additional operations done in the MTP-transfer group may result in increased scarring and explain the lower cosmetic satisfaction.

Metatarsophalangeal-transfer has been linked to improved long-term ulnar growth (Vilkki, 2008) and UL was slightly longer in the MTP-transfer group. As UL depends on stature, previous studies have recommend comparing it to total body height or normative values (Ekblom et al., 2013; Goldfarb et al., 2002; Vilkki, 2008). Owing to the absence of data for participant height and normative values for Finnish populations, we compared UL to the unaffected side in unilateral cases, finding no significant group differences. These results should be interpreted cautiously, given the ongoing growth in some participants and the exclusion of bilateral RLDs.

Complications from the primary procedure were uncommon after both treatments. Most patients underwent additional operations to improve function. Wrist revision operations were more frequent in the MTP-transfer group, probably because of a more invasive treatment approach with extensive reconstructions and a lower threshold for surgical revisions, rather than differences in recurrence of deformity.

The limitations of our study include the small sample size, inclusion of still-growing adolescents, retrospective design and variability in additional operations, which prevent further interpretation of outcomes. The heterogeneity in the presentation of RLD, particularly within Bayne and Klug type IV, complicates the outcome analysis.

Although the PROMs used also assess hand function, finger function, which is critical to hand performance (Van Nieuwenhoven et al., 2023), was not objectively assessed. Although DASH and PRWE have been validated in other contexts, their performance in RLD, or in other congenital disorders, is untested. In paediatric settings, there has been only limited investigation of the shortened version of DASH (QuickDASH) (Kämppä et al., 2024; Quatman-Yates et al., 2013). Proxy response bias may have occurred from assistance provided to participants with cognitive or communication impairments (Sneeuw et al., 2002). Observer bias is possible as treatment groups were assessed separately and partly by investigators involved in their care.

The main strength of this study is the treatment allocation based on geographical referral areas, resembling an expertise-based design covering the entire national population, thus minimizing selection bias.

Patient-rated hand function seems to be similar and relatively good after both procedures. In this series MTP-transfer produced a more consistent and larger AROM of wrist extension–flexion but with poorer cosmetic results than radialization.

## Supplemental Material

sj-docx-1-jhs-10.1177_17531934251391119 – Supplemental material for Radial longitudinal deficiency: long-term outcomes of radialization and vascularized metatarsophalangeal joint transfer Supplemental material, sj-docx-1-jhs-10.1177_17531934251391119 for Radial longitudinal deficiency: long-term outcomes of radialization and vascularized metatarsophalangeal joint transfer by Niko Kämppä, Petra Grahn, Pasi Paavilainen, Yrjänä Nietosvaara and Jarkko Jokihaara in Journal of Hand Surgery (European Volume)
