# Supplementary material for: Radial longitudinal deficiency: long-term outcomes of radialization and vascularized metatarsophalangeal joint transfer
Source: J Hand Surg Eur Vol. 2025 Nov 11;51(4):472–9. doi: 10.1177/17531934251391119 (PMC12967366; doi:10.1177/17531934251391119)
Supplement: sj-docx-1-jhs-10.1177_17531934251391119 – Supplemental material for Radial longitudinal deficiency: long-term outcomes of radialization and vascularized metatarsophalangeal joint transfer [file sj-docx-1-jhs-10.1177_17531934251391119.docx]

**Supplementary table 1**. Course of treatment and complications. K-wire removals not included.

| N:o | B&G type | Primary procedure | | Pollicization | Additional procedures and adverse events | | | | | | | |
| --- | --- | --- | --- | --- | --- | --- | --- | --- | --- | --- | --- | --- |
|  |  |  | Complications |  | Procedure 1 | Complications | Procedure 2 | Complications | Procedure 3 | Complications | Procedure 4 | Procedure 5 |
| 1 | IV | RADIALIZATION | Fracture through ulna physis and partial growth arrest | no | Distractor application | Pain and pintract infection (distraction discontinued) | Ulnocarpal fusion |  |  |  |  |  |
| 2 | IV |  |  | yes | III-IV web space syndactyly separation |  | Ulna osteotomy |  |  |  |  |  |
| 3 | IV |  |  | yes |  |  |  |  |  |  |  |  |
| 4 | IV |  |  | no | Opponensplasty | Collateral ligament reconstruction failure | MCP I chondrodesis and re-opponensplasty |  | Ulnocarpal fusion | Fusion failure |  |  |
| 5 | III |  | K-wire protrusion through skin and k-wire removal | yes |  |  |  |  |  |  |  |  |
| 6 | IV |  |  | yes |  |  |  |  |  |  |  |  |
| 7 | IV |  |  | no |  |  |  |  |  |  |  |  |
| 8 | III |  |  | yes | First web space z-plasty |  |  |  |  |  |  |  |
| 9 | IV |  |  | yes |  |  |  |  |  |  |  |  |
| 10 | IV |  |  | yes |  |  |  |  |  |  |  |  |
| 11 | IV |  | Insufficient K-wire fixation | no |  |  |  |  |  |  |  |  |
| 12 | IV |  |  | no |  |  |  |  |  |  |  |  |
| 13 | IV |  |  | no | Ulna osteotomy and distractor application | Pin loosening (distraction discontinued) | Thumb remnant excision |  |  |  |  |  |
| 14 | IV |  |  | yes |  |  |  |  |  |  |  |  |
| 15 | III |  |  | yes |  |  |  |  |  |  |  |  |
| 16 | III | MTP TRANSFER | Delayed bony union and widening of ulna-graft junction | yes | Ulna osteotomy |  |  |  |  |  |  |  |
| 17 | III |  |  | no |  |  |  |  |  |  |  |  |
| 18 | IV |  |  | yes | Ulna osteotomy |  | Plate removal |  |  |  |  |  |
| 19 | IV |  |  | no |  |  |  |  |  |  |  |  |
| 20 | III |  |  | yes |  |  |  |  |  |  |  |  |
| 21 | IV |  |  | yes |  |  |  |  |  |  |  |  |
| 22 | IV |  |  | no | Distractor application |  | Wrist position supported with K-wires for 7 months | Fracture at MT base and pseudoarthrosis. Eventual fusion seen after 2 years. |  |  |  |  |
| 23 | III |  |  | yes | Reconstruction of a neoradius |  |  |  |  |  |  |  |
| 24 | III |  |  | yes | Reconstruction of neoradius | Radio-ulnar synostosis | Synostosis removal |  |  |  |  |  |
| 25 | IV |  |  | no | Ulna osteotomy and distractor application |  | Flexor slide, FCU pro ECU transposition, distractor application |  |  |  |  |  |
| 26 | IV |  |  | yes |  |  |  |  |  |  |  |  |
| 27 | IV |  |  | no |  |  |  |  |  |  |  |  |
| 28 | IV |  |  | yes |  |  |  |  |  |  |  |  |
| 29 | IV |  |  | yes |  |  |  |  |  |  |  |  |
| 30 | III |  |  | yes | Reconstruction of neoradius | Neoradius nonunion | Fusion of pseudoarthrosis (k-wires) | Nonunion | Re-fusion of pseudoarthrosis (plate) |  | Plate removal | EDQ pro EPL tendon transfer |
| 31 | III |  |  | yes | Reconstruction of radius |  | Ulna epiphysiodesis |  |  |  |  |  |
| 32 | IV |  |  | no |  |  |  |  |  |  |  |  |

B&G type: Bayne and Klug type; MTP-transfer: metatarsophalangeal joint transfer
